# Spontaneous Rupture of Pyometra in a Nonpregnant Young Woman

**DOI:** 10.1155/2017/4572379

**Published:** 2017-02-19

**Authors:** Parvin Mostafa-Gharabaghi, Shima Bordbar, Shabnam Vazifekhah, Mohammad Naghavi-Behzad

**Affiliations:** ^1^Women's Reproductive Health Research Center, Tabriz University of Medical Sciences, Tabriz, Iran; ^2^Department of Obstetrics and Gynecology, Tabriz University of Medical Sciences, Tabriz, Iran; ^3^Reproductive Health Research Center, Department of Obstetrics and Gynecology, Faculty of Medicine, Urmia University of Medical Sciences, Urmia, Iran; ^4^Students' Research Committee, Tabriz University of Medical Sciences, Tabriz, Iran; ^5^Medical Philosophy and History Research Center, Tabriz University of Medical Sciences, Tabriz, Iran

## Abstract

A 40-year-old woman presented with severe vaginal bleeding. Initial workup with an abdominal sonography revealed endometrium for about 3 mm and free fluid in the abdomen. Hemodynamic instability with abdominal pain and free fluid in the abdomen prompted blood transfusion and laparotomy. There were about 1000 cc blood and clots in the abdomen at laparotomy. There was a longitudinal rupture from fundus up to cervix at the left side of the uterus. Tearing was in full thickness from serosa to endometrium. Scar of previous cesarean was transvers and not associated with this tearing. There was not any myomectomy scar.

## 1. Introduction

Known as a rare condition, pyometra is considered as the collection of pus in the uterine cavity [1]. Blockage of the cervical canal secondary to benign or malignant cervical or endometrial lesions and outcomes of their treatments, cervicitis, postvaginal surgery, puerperal infection, and congenital cervical anomaly are the main cause of pyometra [2]. Moreover another rare case is spontaneous perforation of pyometra and subsequent diffuse peritonitis with an incidence of about 0.01%–0.05% [1]. There is a high association between pyometra on one hand and malignancies, risk of perforation, and high mortality rate in a way that clinicians get aware of this disease, especially in postmenopaused women presenting with acute abdomen [3]. A woman was reported who has been treated under a clinical diagnosis of diffuse peritonitis caused by spontaneously perforated pyometra without malignancy.

## 2. Case Presentation

A 40-year-old woman presented with vaginal bleeding to Gynecology Service. Bleeding had started two days earlier and was getting severe. Bleeding had happened after lifting a heavy box and was associated with abdominal pain. The pain was generalized and was associated with vomiting. She had no past medical history. She was using contraception and also she was a pill user. She had 2 children, both delivered via cesarean. She did not have any evidence of sexually transmitted disease. On presentation, her blood pressure was 90/60, she was tachycardic at 96, and her body temperature was 37.2. On examination uterus was about 8–10 weeks, it was tender in palpation, and cervix had a normal appearance with a heavy bleeding. Her abdomen was generally tender.

Differential diagnosis consisted of ectopic pregnancy (EP), abortion, rupture of an ovarian cyst, endometrial cancer, uterine sarcoma, degenerating myoma, pelvic inflammatory disease (PID), and rupture of the cesarean scar.

**Figure 1:**
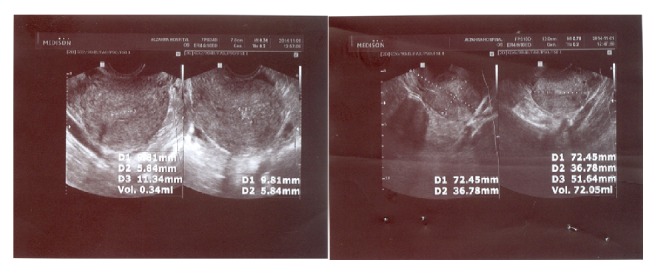
Abdominal ultrasonography view of patient before any treatment protocol.

*Investigations*. Due to vaginal bleeding and abdominal pain we first checked Beta-Human Chorionic Gonadotropin (BHCG) for ruling out pregnancy. BHCG was negative. Laboratory investigations revealed hemoglobin of 6.4 g/dL. In the context of severe vaginal bleeding and abdominal pain an urgent abdominal sonography was performed which showed that the endometrium was about 3 mm and there was remarkable free fluid in the abdomen (Figure 1).

## 3. Approach and Treatment Protocol

Considering abdominal pain, low blood pressure, and remarkable free fluid in the abdomen, we began blood transfusion and did laparotomy. We transfused 3 units of packed-cell.

**Figure 2:**
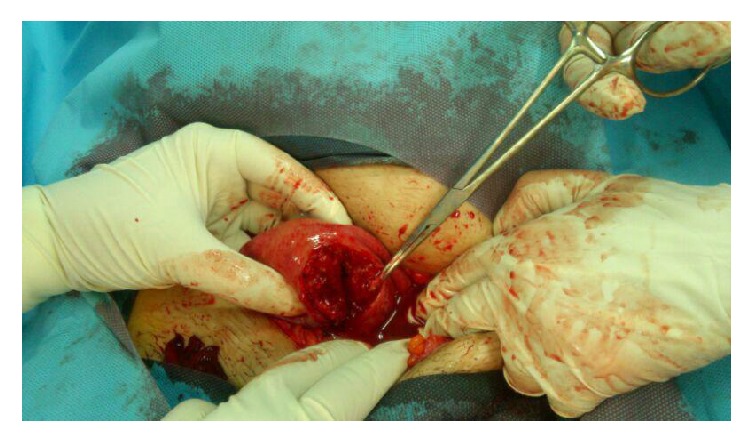
Rupture view of fundus up to cervix at the left side of the uterus in operation room.

There were about 1000 cc blood and clots in the abdomen. There was a longitudinal rupture from fundus up to cervix at the left side of the uterus. Tearing was in full thickness from serosa to endometrium (Figure 2). Previous cesarean scar was intact.

The patient did not have any desire for the next pregnancy, so we did hysterectomy and saved both of ovaries.

## 4. Outcome and Follow-Up

After surgery and recovery, the patient was transferred to the ward. After three days, she discharged from hospital with stable vital sign and good status. Uterus pathology revealed no specific findings and only inflammation was seen in investigated tissues which can be in favor of spontaneous rupture of pyometra as the main reason.

## 5. Discussion

Uterine rupture is defined as disruption or tear of the myometrium and serosa of uterus [4]. Spontaneous rupture of the uterus is an uncommon condition occurring mainly in elderly postmenopausal females and results when natural drainage of the uterine cavity is compromised. But documented finding in pregnant women and also the occurrence of spontaneous uterus rupture in a nongravid woman are much rarer [5, 6].

Previous cesarean scar or myomectomy, trauma, grand-multiparity, uterine anomaly or injudicious use of oxytocin or prostaglandin, and neglected labor are some of the predisposing factors for uterine rupture during labor [7–9].

Most ruptures occur in women who have had a previous transmyometrial surgical incision, typically for cesarean delivery [10].

In a study of uterine ruptures in Netherlands, the incidence of rupture in unscarred and scarred uteri was 0.7 and 5.1 per 10,000 deliveries, respectively [11]. A study from the United States reported rupture of the unscarred uterus in 4.54 per 100,000 deliveries or about 1 in 22,000 deliveries [10]. The incidence of rupture in both scarred and unscarred uteri has increased during recent decades [12].

Vyas et al. reported that multidetector computed tomography (CT) with sagittal and coronal reformatted images may aid preoperative diagnosis of the ruptured pyometra [13]. As a result of reported studies, 33% of patients were diagnosed correctly using CT or magnetic resonance imaging [14, 15].

In this case after lifting a heavy box, rupture of uterus occurred. The occurrence of the rupture is associated with past surgical history of cesarean section.

The signs and symptoms of uterine rupture, largely depending on timing, site, and extent of uterine defect, are severe hemorrhage, palpable fetal parts, loss of uterine contractility and rarely blood stained urine, appearance of placenta at vulva, and prolapsing of loops of gut into vagina [16, 17].

Some signs and symptoms are common in patients. Abdominal pain is reported to occur in up to 13–60% of cases, vaginal bleeding in 11–67% of patients, and hemorrhagic shock in up to 46% of patients [18, 19].

Intensive antibiotic therapy is considered important for a favorable outcome because these patients easily develop generalized peritonitis followed by septic shock [20].

The cases of spontaneously type pyometra without associated with malignancy have better prognosis as compared to those cases that are associated with malignancy. This conclusion is based on the fact that 73% of nonmalignant cases had a favorable prognosis for survival [21].

The uterine rupture is most appropriately diagnosed on the basis of standard signs and symptoms. Considering short time available to diagnose uterine rupture, time-consuming diagnostic methods and sophisticated imaging modalities have a limited utility [17].

## References

[1] Geranpayeh L., Fadaei-Araghi M., Shakiba B. (2006). Spontaneous uterine perforation due to pyometra presenting as acute abdomen. *Infectious Diseases in Obstetrics and Gynecology*.

[2] Lee S.-L., Huang L.-W., Seow K.-M., Hwang J.-L. (2007). Spontaneous perforation of a pyometra in a postmenopausal woman with untreated cervical cancer and “forgotten” intrauterine device. *Taiwanese Journal of Obstetrics & Gynecology*.

[3] Jeon H., Shin H., Kim I., Chung H., Chung D. (2012). Spontaneous uterine perforation of pyometra presented as an acute abdomen: a case report. *Korean Journal of Obstetrics & Gynecology*.

[4] Kiseli M., Artas H., Armagan F., Dogan Z. (2013). Spontaneous rupture of uterus in midtrimester pregnancy due to increased uterine pressure with previous laparoscopic myomectomy. *International Journal of Fertility & Sterility*.

[5] Kieser K. E., Baskett T. F. (2002). A 10-year population-based study of uterine rupture. *Obstetrics & Gynecology*.

[6] Weng L. C., Menon T., Hool G. (2013). Spontaneous rupture of the non-gravid uterus. *BMJ Case Reports*.

[7] Berghella V., Airoldi J., O'Neill A., Einhorn K., Hoffman M. (2009). Misoprostol for second trimester pregnancy termination in women with prior caesarean: a systematic review. *BJOG*.

[8] Henderson C. E., Hana R. G., Woroch R., Reilly K. D. (2010). Short interpregnancy interval and misoprostol as additive risks for uterine rupture: a case report. *Journal of Reproductive Medicine for the Obstetrician and Gynecologist*.

[9] Latika S. (2006). A 10 year analysis of uterine rupture at a teaching institution. *The Journal of Obstetrics and Gynecology of India*.

[10] Gibbins K. J., Weber T., Holmgren C. M., Porter T. F., Varner M. W., Manuck T. A. (2015). Maternal and fetal morbidity associated with uterine rupture of the unscarred uterus. *American journal of obstetrics and gynecology*.

[11] Zwart J. J., Richters J. M., Öry F., De Vries J. I. P., Bloemenkamp K. W. M., Van Roosmalen J. (2009). Uterine rupture in the Netherlands: a nationwide population-based cohort study. *BJOG*.

[12] Al-Zirqi I., Stray-Pedersen B., Forsén L., Daltveit A.-K., Vangen S. (2016). Uterine rupture: trends over 40 years. *BJOG: An International Journal of Obstetrics & Gynaecology*.

[13] Vyas S., Kumar A., Prakash M., Kapoor R., Kumar P., Khandelwal N. (2009). Spontaneous perforation of pyometra in a cervical cancer patient: a case report and literature review. *Cancer Imaging*.

[14] Chan K.-S., Tan C.-K., Mak C.-W., Chia C.-C., Kuo C.-Y., Yu W.-L. (2006). Computed tomography features of spontaneously perforated pyometra: a case report. *Acta Radiologica*.

[15] Izumi J.-I., Hirano H., Yoshioka H., Takisawa J. (2010). Computed tomography findings of spontaneous perforation of pyometra. *Japanese Journal of Radiology*.

[16] Khanum Z., Lodhis K. (2004). Emergency obstetric hysterectomy: a life saving procedure. *Annals of King Edward Medical University*.

[17] Qudsia Q., Akhtar Z., Kamran K., Khan A. H. (2012). Woman health; uterus rupture, its complications and management in teaching hospital Bannu, Pakistan. *Maedica*.

[18] Gurudut K. S., Gouda H. S., Aramani S. C., Patil R. H. (2011). Spontaneous Rupture of Unscarred Gravid Uterus. *Journal of Forensic Sciences*.

[19] Mallah F., Eftekhar T., Naghavi-Behzad M. (2013). Spontaneous rupture of pyometra. *Case Reports in Obstetrics and Gynecology*.

[20] Ikeda M., Takahashi T., Kurachi H. (2013). Spontaneous perforation of pyometra: a report of seven cases and review of the literature. *Gynecologic and Obstetric Investigation*.

[21] Saha P. K., Gupta P., Mehra R., Gael P., Huria A. (2008). Spontaneous perforation of pyometra presented as an acute abdomen: a case report. *MedGenMed Medscape General Medicine*.

